# Effect of Hydrophobic Fumed Silica on Bending Strength of Sodium Silicate-Bonded Sand Cores

**DOI:** 10.3390/ma17235714

**Published:** 2024-11-22

**Authors:** Yunbo Li, Huarui Zhang, Jiulong Chen, Ting Xiang, Ying Cheng, Hu Zhang

**Affiliations:** 1 Ningbo Institute of Technology (NIT), Beihang University, Ningbo 315000, China; d00992@buaa.edu.cn (Y.L.); xiangting0922@163.com (T.X.); 2 School of Materials Science and Engineering, Beihang University, Beijing 100191, China; king12311231@buaa.edu.cn (J.C.); cying@buaa.edu.cn (Y.C.)

**Keywords:** hydrophobic fumed silica, sodium silicate-bonded sand, aluminum alloy castings, bonding bridges

## Abstract

Inorganic sand cores involving sodium silicate binder and microsilica have environmental advantages during the casting process of aluminum alloy. Nevertheless, the bending strength of sodium silicate-bonded sand (SSBS) needs to be further improved. In this research, the effect of hydrophobic fumed silica on the bending strength of sand cores was studied. The experimental results revealed that hydrophobic fumed silica with the addition of 0.050 wt.% can be adopted as an optimal modifier to enhance the bending strength of SSBS. According to scanning electron microscope and spectroscopy techniques, dense bonding bridges and a complex Si–O–Si network containing specific silicon molecules with a silicon atom bonded to three other silicon atoms contribute to the excellent bending strength, with a 53.1% increase in cold strength (24 h) compared to a commercial sample of a modified sand core. Meanwhile, the newly formed Si–O–Al chemical bond plays a crucial role in increasing the bending strength of sand cores.

## 1. Introduction

With increasingly stringent environmental regulations, the foundry industry is facing ever more complex challenges. Aluminum alloy has been widely used in aerospace, automobiles, electronics, weapons and other fields due to its low density, high specific strength and stiffness and excellent casting performance. Sand cores are used to form inner cavities and holes, which play a decisive role in producing metal castings with complicated geometries, especially for the production of aluminum alloy castings [1,2]. For now, the casting industry commonly uses organic binder sand, which causes a large number of volatile organic compounds to be released during the low-pressure casting process of aluminum alloy, which endangers the health of on-site producers and causes environmental pollution [3,4]. However, the raw materials of organic binders are mainly the by-products of agricultural chemicals and petroleum products. Kmita et al. reported that resin binder will generate CO, CO_2_, NH_3_, H_2_O, phenol, CH_4_ and other compounds at a lower pyrolysis temperature. After heating at higher temperatures, phenol, along with its methyl and ethyl derivatives and benzene releases, accounts for a large amount of volatile organic compound emissions [5,6]. Under this circumstance, a new inorganic binder sand has emerged in the foundry industry in response to the increasing demand for solving the environmental problems in the production of aluminum alloy castings with complex shapes, namely a two-component binder system including modified sodium silicate solutions and powder curing agents, for instance, microsilica [7,8,9,10,11].

In recent years, large-scale research efforts have been devoted to the development of inorganic binder sand. However, its lower room-temperature strength hinders practical application. The research into the enhancement of bending strength has mainly focused on the modification of sodium silicate binder [12,13]. For example, Lai et al. studied the influence of the irreversible reaction between borax and polyvinyl alcohol, thus improving the moisture absorption resistance of sodium silicate-bonded sand [14]. For instance, Song et al. showed that the addition of disodium polyvinylpyrrolidone, lithium tetraborate and hydrogen phosphate could obviously enhance the tensile strength of SSBS through boosting the bonding bridges and making them tighter and smoother at room temperature [15]. Due to the limitations of the conventional binder system, Jung et al. developed a new ternary-phase binder system incorporating alumina precursors to enhance the fracture strength of molds for casting [16]. The further improvement of the strength is still limited. Moreover, research on enhancing the strength by using powder curing agents is rarely reported, especially for microsilica. It is known that the main component of microsilica powder is amorphous SiO_2_, which can significantly improve the bonding properties of inorganic binder sand. Song’s group reported that highly active micro-silicon powder and liquid sodium silicate binder undergo polymerization dehydration and a cross-linking curing reaction in an alkaline environment, thus forming a dense three-dimensional network structure which can significantly improve the hardening rate and greatly improve the water resistance of the sand core [17].

The mechanical properties of the new silicate inorganic binder sand are closely associated with its molecular structure and interfacial characteristics before and after curing. In this study, performance tests and experimental characterizations show that hydrophobic fumed silica can significantly enhance the bending strength of SSBS, with a 53.51% increase in cold strength (24 h) compared to a commercial powder sample of a modified sand core. In addition, the corresponding mechanism of the addition of hydrophobic fumed silica on the bending strength of sodium silicate-bonded sand cores and the interfacial characteristics were analyzed.

## 2. Materials and Methods

### 2.1. Materials

The microsilica generally applied in the foundry industry was selected for the experiment, and its composition is shown in Table 1. The hydrophobic fumed silica was purchased from Ecosoli, Shandong, China. Meanwhile, the Inotec Promotor WJ 4000 was chosen as a commercial standard sample from ASK Chemicals, Pune, India. Typically, a sodium silicate binder with a constant solid content and Si/Na ratio was used as a modified sodium silicate solution, the same as water glass.

### 2.2. Physical Characterizations

Scanning electron microscope (SEM) images were recorded on a GeminiSEM G360 operated at 20 KV equipped with an EDS detector (Carl Zeiss AG, Oberkochen, Germany). Powder X-ray diffraction patterns were obtained using a Bruker D8 Advance X-ray diffractometer using a Cu Kα radiation source (Bruker, Billerica, MA, USA). The optical images for the sand cores before and after fracturing were determined by an ultra-depth of field 3D microscope system (HIROX, Tokyo, Japan).

The composition of the microsilica was determined by an X-ray fluorescence spectrometer (XRF) on an S8 TIGER (Bruker, Billerica, MA, USA). An XPS (X-ray photoelectron spectroscopy) spectrum was obtained with XPS (Kratos, Manchester, UK) using AlKα radiation on AXIS Supra+. The structural analysis of the powder sample was analyzed through spectrum methods, Fourier transform infrared (FTIR) spectroscopy on a Nicolet IS50 (Thermo Scientific, Waltham, MA, USA).

### 2.3. Experimental Produce

**Figure 1:**
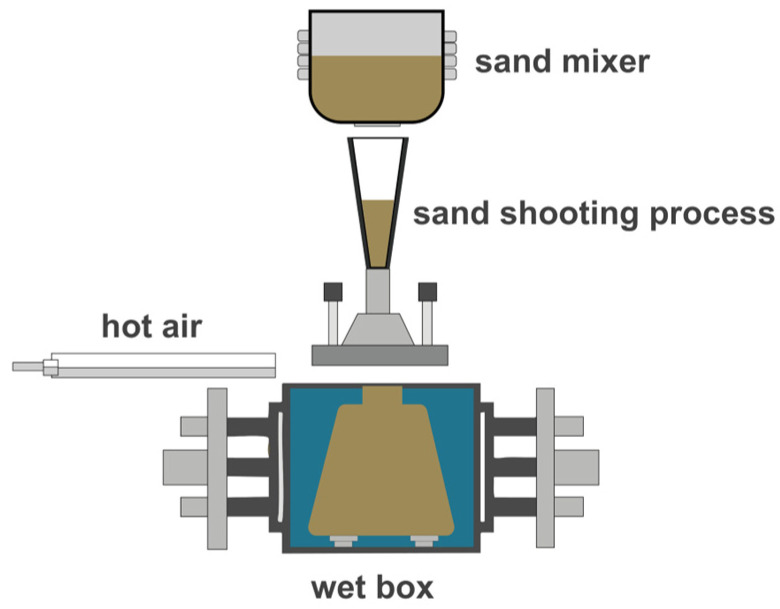
Schematic of the preparation process of inorganic sand cores for casting.

**Figure 2:**
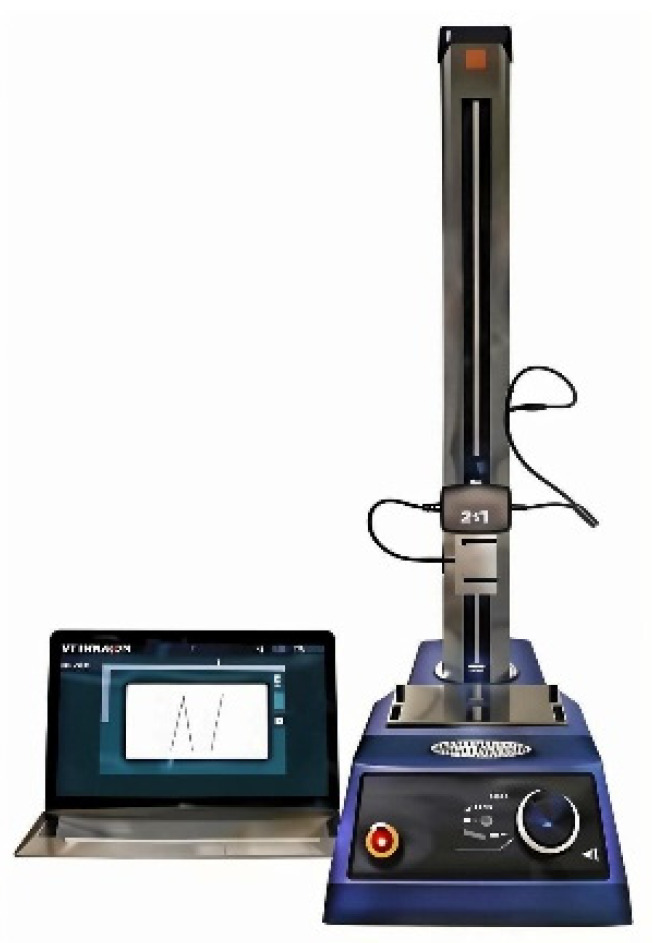
Schematic of physical property analyzer (TMS-plus).

**Figure 3:**
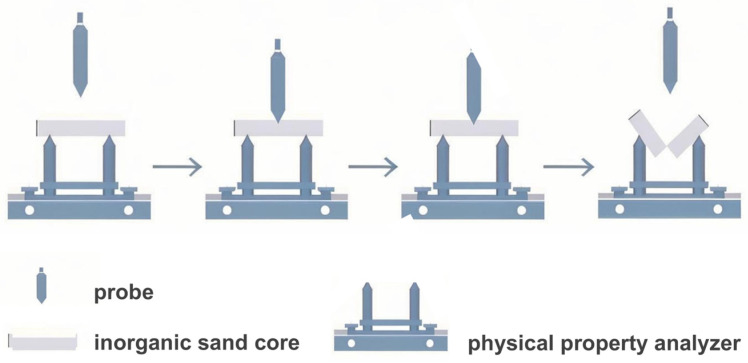
Schematic of the three-point bending of inorganic sand cores curved.

Before the experiments, the mixed powder was prepared by the addition of hydrophobic fumed silica with different contents (0.025, 0.050, 0.075 and 0.100 wt.%) into microsilica, and the mixed powder and modified sodium silicate solutions were mixed in a ratio of 1:4. A mixture of microsilica, modified sodium silicate solution and AFS50 silica sand was prepared with a ratio of 1:2:100. After being mixed evenly, the mixture was injected into a mold with dimensions of 173.36 mm × 22.36 mm × 22.36 mm, and the mixture was heated to 160–220 °C for 40–60 s to fully cure the inorganic sand cores; then, high-pressure hot air was blown into the mold to form sand cores, as depicted in Figure 1. The product after dehydration and condensation was used as a sample and stored for <1 min, 1 h and 24 h at 30–45% RH and 20–25 °C before the test. A physical property analyzer (Food Technology Corporation, Sterling, VA, USA) was used to analyze the bending strength of the sand core by a three-point bending fixture, as shown in Figure 2. Figure 3 shows the three-point test process for SSBS. The vertices of the three probes were in a straight line. When the zero point of displacement was placed, the sample was placed. The upper probe pressed towards the test sample after touching the table of the sample. After the surface of the sample was touched, the sample was pressed down. When the sample was squeezed and broken, the probe returned to the specified height [18,19].

Each sample was measured at least five times to ensure reproducibility and repeatability, and the average value was chosen as the final bending strength. The bending strength could be deduced from the following equation [20,21]:

(1) $$\sigma =\frac{3PL}{2WH}$$

where *σ* stands for the bending strength, MPa; *P* represents the load, N; *L* stands for the fulcrum span, 151 mm; *W* represents the sample width, 22.26 mm; and *H* stands for the sample thickness, 22.36 mm.

## 3. Result and Discussion

### 3.1. Morphological and Structural Characterizations

Hydrophobic fumed silica is obtained by the surface modification of hydrophilic silica with a silane coupling agent. After modification, a large number of hydroxyl groups on the surface of silica particles are replaced by hydrophobic groups, resulting in excellent hydrophobicity and a low hygroscopic property [22,23]. The purity of fumed silica is as high as 99%, and the fumed silica has a huge specific surface area, good dispersion and high activity. Based on the excellent hydrophobicity of hydrophobic fumed silica, hydrophobic fumed silica was selected as a powder accelerator to improve the bending strength performance of sodium silicate-bonded sand cores under different addition amounts.

**Figure 4:**
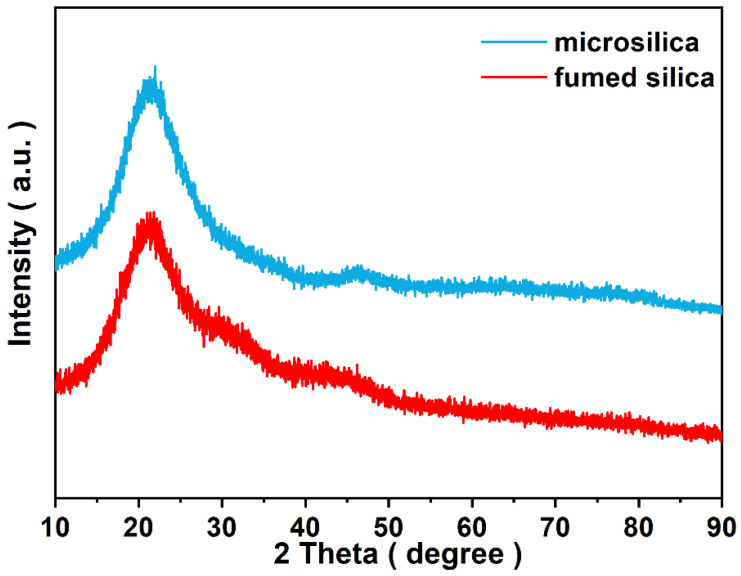
Powder XRD patterns of microsilica and powder with the addition of fumed silica.

**Figure 5:**
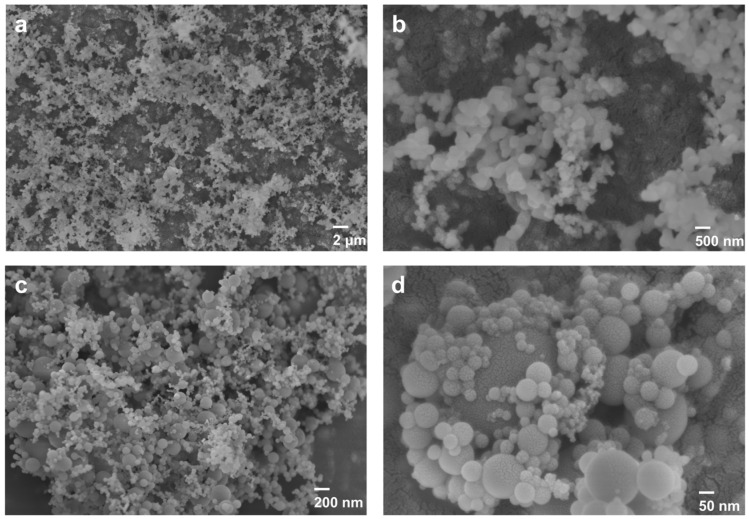
SEM images of fumed silica (**a**,**b**) and microsilica (**c**,**d**).

The powder X-ray diffraction (XRD) patterns in Figure 4 indicate that the main crystalline structure of microsilica is amorphous silicon dioxide due to the existence of the broad peak at around 21.5° and 46.4° [24]. It can be seen unambiguously that in the red line of the XRD patterns, the main characteristic broad peaks for the sample with the addition of hydrophobic fumed silica have no significant changes. The element composition of microsilica was probed by an XRF measurement. The main component of microsilica powder is silicon dioxide, which is consistent with the XRD results. Meanwhile, the SEM images in Figure 5 reveal that microsilica and hydrophobic fumed silica are both well-dispersed round sphere-shaped particles.

### 3.2. Bending Strength of Sand Core

The bending strength of all the samples was determined by the three-point test method, as shown in Figure 3. Sand cores with the addition of different contents of hydrophobic fumed silica were prepared to assess the optimal addition amount. Meanwhile, the sand cores of all the samples were stored for <1 min, 1 h and 24 h to test the bending strength and evaluate storage stability.

**Figure 6:**
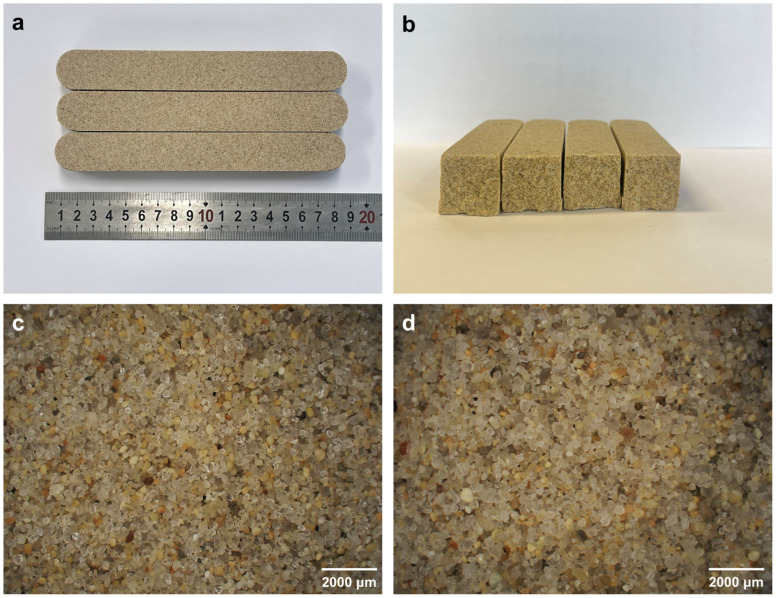
Digital photographs of standard three-point bending specimens for inorganic sand core before (**a**) and after fracture (**b**); optical images of standard three-point bending specimens for inorganic sand core before (**c**) and after fracture (**d**).

In order to completely characterize the morphological characteristics and particle distribution of the sand cores before and after fracturing, the optical images were determined by an ultra-depth of field 3D microscope system, as shown in Figure 6. As shown in Figure 6, it can be clearly found that the sand cores have the same morphology and uniform distribution of particles before and after fracturing, and the digital photographs of standard three-point bending specimens for inorganic sand cores have no obvious change before and after fracturing, which proves the successful preparation of the sand cores.

**Figure 7:**
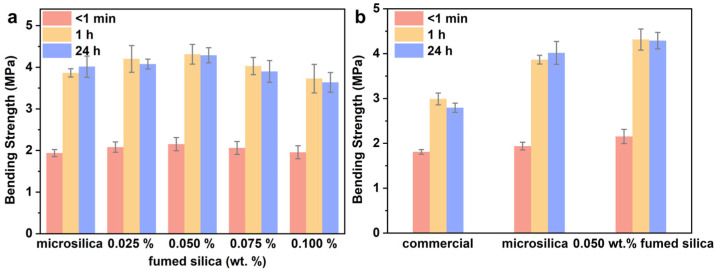
(**a**) Bending strength of inorganic sand core cured by microsilica and the different additions of fumed silica (0.025, 0.050, 0.075 and 0.100 wt.%); (**b**) bending strength of inorganic sand core cured by microsilica, the 0.050 wt.% addition of the fumed silica and the commercial sample.

As depicted in Figure 7a, the sand core modified by fumed silica with the addition of 0.050 wt.% exhibits the highest bending strength among all the samples tested (0.025, 0.050, 0.075 and 0.100 wt.%). With the increase in the amount of fumed silica, the strength of the sand core increases first and then decreases, indicating that a small amount of fumed silica can improve the bending strength properties of the sand core. The thermal strength (<1 min) of the sand core modified by fumed silica with the addition of 0.050 wt.% is higher compared to that of the sand core modified by microsilica and the commercial standard sample, while the sand core modified by fumed silica with the addition of 0.050 wt.% shows an 18.95% higher bending strength compared to sand core modified by the commercial standard sample. Meanwhile, for the sand core modified by fumed silica with the addition of 0.025 wt.% and 0.075 wt.%, the thermal strength (<1 min) of the sand core is still higher compared to that of the sand core modified by microsilica and the commercial standard sample, showing a 14.97% and 13.81% higher bending strength compared to that of the sand core modified by the commercial standard sample. As for the sand core modified by fumed silica with the addition of 0.050 wt.%, the cold strength (1 h) is 4.314 MPa, which is much greater than that of the sand core modified by microsilica (3.865 MPa) and commercial standard sample (2.991 MPa), as depicted in Table 2. It is worth noting that the sand core modified by fumed silica with the addition of 0.050 wt.% shows a 44.23% and 11.62% higher bending strength compared to that of the sand core modified by the commercial standard sample and microsilica, respectively. Furthermore, for the sand core modified by fumed silica with the addition of 0.025 wt.%, the cold strength (1 h) of the sand core is still higher compared to that of the sand core modified by microsilica and the commercial standard sample, which shows a 40.49% higher bending strength compared to that of the sand core modified by the commercial standard sample. After being stored for 24 h at 30–45% RH, the bending strength of the sand core modified by fumed silica with the addition of 0.050 wt.% still remains high (4.077 MPa) compared to the bending strength after storage for 1 h, which is about 53.51% higher compared to that of the sand core modified by the commercial standard sample. In the meantime, for the sand core modified by fumed silica with the addition of 0.025 wt.% and 0.075 wt.%, the bending strength of the sand core after storage for 24 h at 30–45% RH is still higher compared to that of the sand core modified by the commercial standard sample, which shows a 45.92% and 39.58% higher bending strength compared to that of the sand core modified by the commercial standard sample. However, the bending strength after storage for 24 h at 30–45% RH for the sand core modified by fumed silica with the addition 0.075 wt.% is lower compared to that of the sand core modified by microsilica. The results indicate that the optimal addition of the hydrophobic fumed silica is beneficial to the enhancement of the bending strength. Based on the data of bending strength obtained under laboratory conditions, it can be seen that the thermal strength and the cold strength of the sand core modified by fumed silica with the addition of 0.050 wt.% can reach the industrial usage index. Thus, it can be further applied to the production of aluminum castings. Meanwhile, the introduction of substances containing hydrophobic groups and amphoteric oxides is also beneficial to improve the bending strength of inorganic sand cores based on the above results.

Hydrophobic fumed silica has a larger specific surface area, and a small amount of dissolved SiO_2_ has more uniform contact with the binder. Therefore, under alkaline conditions, a part of the Si-O-Si bond is hydrolyzed to form Si-OH, and more silicic acid gels are formed by condensation under heating, which is beneficial for improving the modulus of silicate to a certain extent. Therefore, the addition of hydrophobic silica can moderately improve the bending strength of sand cores.

### 3.3. Mechanism Analysis

**Figure 8:**
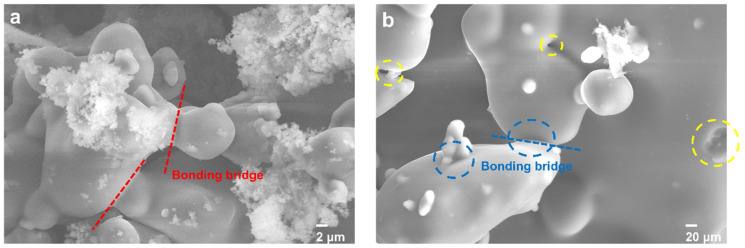
Microscopic morphology of inorganic sand core cured by microsilica (**a**) and sample with the addition of 0.050 wt.% fumed silica (**b**); the yellow dotted circles are the holes, and the blue dotted circles are the cracks.

The surface morphology of the inorganic sand core cured by microsilica and fumed silica with the addition of 0.050 wt.% was found for the surface of the bonding bridges. It can be observed that the surface of the inorganic sand core was surrounded by the mixture of the microsilica and modified water glass and formed a bonding bridge, which is a three-dimensional network structure formed by the dehydration condensation reaction of sodium silicate solution and microsilica under the condition of heating [17]. The strength of the inorganic sand core depends on the difference between the cohesion strength of the bonding bridge. As shown in Figure 8a, it can be found that the surface of the bonding bridges of the sand core modified by the fumed silica with the addition of 0.050 wt.% was comparatively flat and dense, without the obvious cracks or holes. In contrast, Figure 8b shows the holes that emerged in the bonding bridge of the inorganic sand core modified by the microsilica, corresponding to the decline in the bending strength of the inorganic sand core. Therefore, the strength of the sand core modified by the fumed silica with the addition of 0.050 wt.% is significantly enhanced. This may be due to the presence of a small content of hydroxyl on the surface of hydrophobic fumed silica; thus, the reaction speed is relatively slow, while the combined water generated by the condensation reaction can be discharged in time, so the internal bond is dense and uniform.

**Figure 9:**
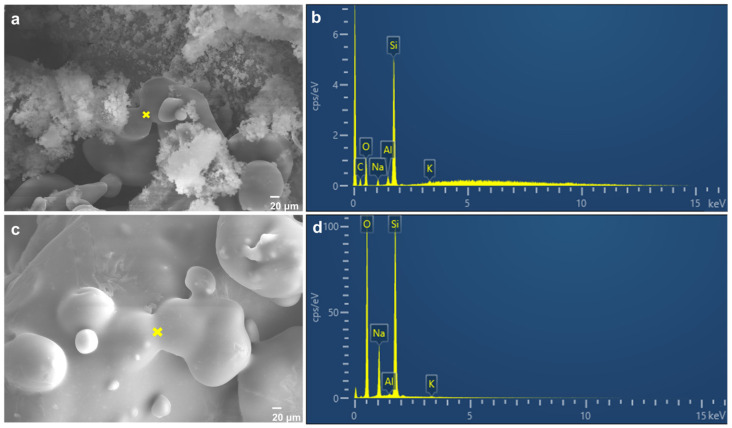
Point scanning of SEM images for inorganic sand core cured by sample with the addition of 0.050 wt.% fumed silica (**a**,**b**) and microsilica (**c**,**d**), where point scanning is at the yellow intersection position.

The element compositions of the inorganic sand core cured by microsilica and fumed silica with the addition of 0.050 wt.% were analyzed with SEM and EDS by point scanning in the bonding bridge. As shown in Figure 9, the main elements for the sample modified by microsilica and fumed silica with the addition of 0.050 wt.% were Si atoms and O atoms. However, it is worth mentioning that the Al element also exists in both samples. To further compare the contents of Al and Si elements, the mass fractions of the Al and Si elements after normalization for the sample modified by fumed silica with the addition of 0.050 wt.% are 1.92% and 32.14%, respectively, while the mass fractions of the Al and Si elements after normalization for the sample modified by microsilica are 0.18% and 27.49%, respectively, as shown in Table 3. Furthermore, the Al/Si ratio for both samples was calculated; the Al/Si ratios of the inorganic sand cores cured by microsilica and fumed silica with the addition of 0.050 wt.% are 0.0065 and 0.060. It is obvious that the difference between the two samples can be seen in the content of Al. This may be the reason for the difference in the bending strength of the sand cores. In the alkaline silicate solution, Al element and OH^−^ combined undergo the hydrolysis reaction. As the concentration of OH^−^ in the solution decreased, the hydrolysis reaction of sodium silicate proceeded in the direction of producing silicic acid, and more silicic acid was obtained. At the same time, Si(OH)_4_ will react with OH^−^ to form a large number of silicic acid anions, and aluminate ions in the solution react with silicic acid anions to form aluminum–silicon mixed ligand complexes. At this time, the newly formed Si-O-Al bond, together with Si-O-Si, forms a silica tetrahedral group with higher cohesion strength in three-dimensional space, which increases the bending strength of the sand core [25,26]. Thus, the newly formed Si-O-Al chemical bond plays a crucial role in increasing the bending strength of sand cores.

**Figure 10:**
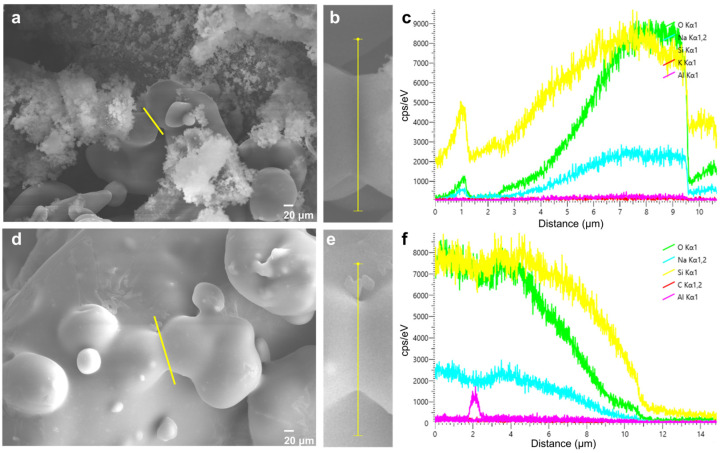
Line scanning of SEM images for inorganic sand core cured by sample with the addition of 0.050 wt.% fumed silica (**a**,**b**) and microsilica (**d**,**e**); linear scanning element distribution for inorganic sand core cured by sample with the addition of 0.050 wt.% fumed silica (**c**) and microsilica (**f**), where line scanning is at the yellow line position.

In order to further prove the interaction between Si and Al elements, an SEM line scanning test of the bonding bridge of the inorganic sand core cured by microsilica and fumed silica with the addition of 0.05 wt.% was performed. As shown in Figure 10, the main elements for the sample modified by microsilica and fumed silica with the addition of 0.050 wt.% were still Si atoms and O atoms. Meanwhile, the Al element also exists in both samples. To further compare the contents of Al and Si elements, the mass fractions of the Al and Si elements after normalization for the sample modified by fumed silica with the addition of 0.050 wt.% are 1.41% and 35.83% as shown in Table 4, respectively, while the mass fractions of the Al and Si elements after normalization for the sample modified by microsilica are 0.45% and 25.23%, respectively, in accordance with the point scanning result. Furthermore, the Al/Si ratio for both samples was calculated; the Al/Si ratios of the inorganic sand core cured by microsilica and fumed silica with the addition of 0.050 wt.% are 0.039 and 0.018. It is obvious that the difference between the two samples can be seen in the content of Al. In an alkaline solution, sodium silicate will be hydrolyzed to form silicate acid and a large amount of OH^−^, and the Si-O molecular bonds will break. Part of the silicon and aluminum elements dissolve from the inside and surface of the particles into a sodium silicate solution, and more silica and aluminum elements react with sodium silicate to produce a gel, resulting in a Si-O-Al bond and more Si-O-Si bonds. With the increase in Si-O-Si bond content, the number of polysiloxy tetrahedrons in the bonding network increases, and the number of polymers with low molecular weight decreases, thus improving the cohesion strength and stability of the bonding network due to the introduction of Al [27,28].

**Figure 11:**
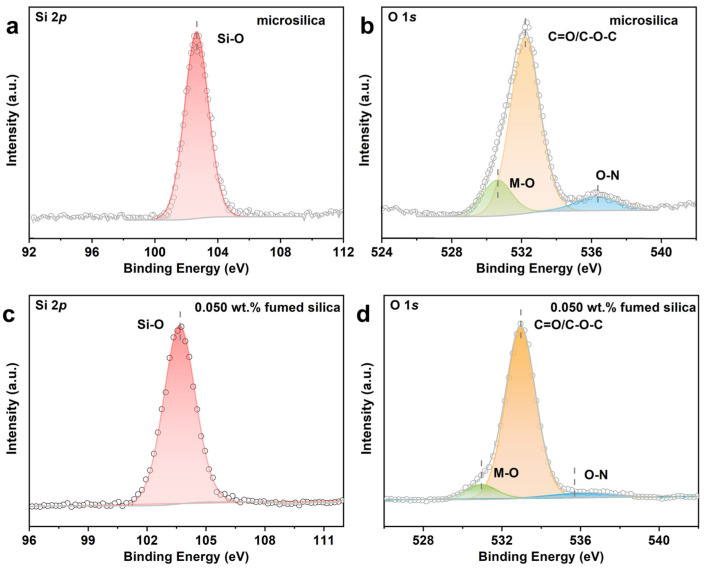
XPS spectrum of Si 2*p* (**a**,**c**) and O 1*s* (**b**,**d**) for the samples cured by water glass powder curing agent: microsilica and sample with the addition of 0.050 wt.% fumed silica.

XPS was conducted to study the surface elemental composition and chemical state of solid silicate bonding bridges for samples cured by microsilica powder and fumed silica with the addition of 0.050 wt.%. The Si 2*p* and O 1*s* spectra were convoluted according to the procedure reported in the literature. As shown in Figure 11a,b, compared with the sample modified by the microsilica, the binding energy of Si 2*p* for the sample modified by fumed silica with the addition of 0.050 wt.% is increased by 0.9 eV, suggesting electron transfer from Si to O [29]. As for the O 1*s* spectrum, three groups of peaks are observed. The peaks for the sample modified by fumed silica with the addition of 0.050 wt.% located at 530.9 eV, 532.9 eV and 536.3 eV can be assigned to M(Si)-O, C=O/C-O-C and M-N peaks, respectively [30,31,32]. Meanwhile, the O 1*s* spectrum peaks for the sample modified by the microsilica can be also assigned to M(Si)-O, C=O/C-O-C and M-N peaks, respectively, which indicates a similar chemical environment for both samples. In the silicate bonding bridge, a coupling reaction occurs between the adhesives, and the polar functional groups in the system will combine with the silicic acid colloidal particles in sodium silicate solution, which is related to foreign carbon and aliphatic carbon in the sol-gel network, thus forming the carbon-chain polymer on the silica composite surface, which is consistent with the literature reports [33,34,35].

The Si-O(H) on the surface of the silicate and the Si-O(H) on the surface of the microsilica powder undergo a dehydration condensation reaction to form a new Si-O-Si bond between the sand particles, while sodium silicate solution and microsilica powder are cured to form a bonding bridge [36]. When more and more Si atoms in the bonding bridge bond with each other, the cohesion strength of the bonding bridge increases; thus, the strength of the inorganic sand core increases.

**Figure 12:**
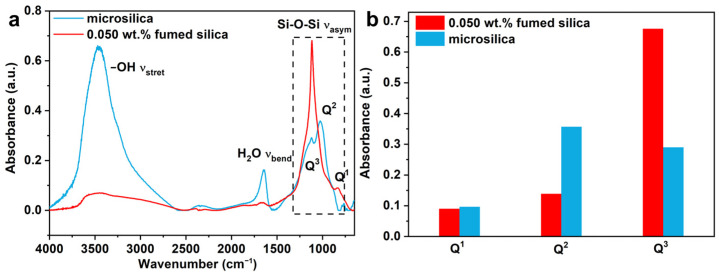
(**a**) FTIR absorbance spectrum of the samples cured by water glass–microsilica: microsilica and sample with the addition of 0.050 wt.% fumed silica. (**b**) Compared intensity of Q^1^, Q^2^ and Q^3^ for each sample.

To further corroborate the chemical structure of the composites of microsilica and modified water glass, FTIR was conducted and compared with the different samples. As shown in Figure 12a, the spectra of the sample modified by microsilica and fumed silica with the addition of 0.050 wt.% generally show the same nature. It can be seen that there are bands belonging to OH stretching vibrations (OH υ_stret_) around 3550 cm^−1^, H_2_O molecule stretching vibrations (H_2_O υ_bend_) around 1640 cm^−1^ and asymmetric stretching vibrations of Si-O-Si (H_2_O υ_bend_) in range of 800–1300 cm^−1^, showing good agreement with the previously reported literature data [37,38,39]. In addition, bridging oxygen atoms are randomly distributed in the complex Si-O-Si network; the degree of polymerization for Q^(n)^ species is applied to specify the density of the number of Si-O-Si bonds, where Q is SiO_4_ tetrahedral units and n represents the number of bridging oxygen (0 ≤ n ≤ 4) [40,41,42].

As known from Figure 12a, the broad peak can be attributed to the consistency of Q^1^ (ca. 892.7 cm^−1^), Q^2^ (ca. 1026 cm^−1^) and Q^3^ (ca. 1121 cm^−1^) species. Each peak position located in the range of 800–1300 cm^−1^ was unchanged, while the intensity changed obviously. Figure 12b shows the detailed intensity of Q^n^ species in the form of a bar chart. With the modification of fumed silica by the addition of 0.050 wt.%, the intensity of the Si-O-Si bond significantly enhanced in comparison with the microsilica, especially for Q^3^ species, indicating the formation of the dense Si-O-Si network in the bonding bridge. Therefore, the strength of the inorganic sand core is enhanced.

## 4. Conclusions

In this work, performance tests and experimental characterizations have demonstrated that hydrophobic fumed silica with the addition of 0.05 wt.% can significantly enhance the bending strength of SSBS by promoting the condensation reaction of microsilica and water glass. According to the experimental analysis, the addition of the hydrophobic fumed silica plays a pivotal role in forming a stable and complex three-dimensional Si-O-Si network structure, thus increasing the bending strength of the bonding bridge between silica sand particles. Meanwhile, the newly formed Si-O-Al chemical bond plays a crucial role in increasing the bending strength of sand cores. The study identifies the optimal amount of fumed silica and analyzes the curing mechanism of the new type of silicate-bonded sand, thus revealing the mechanism by which the mechanical properties of SSBS are improved. This research provides a theoretical basis for optimizing the composition of powder curing agents and enhancing the comprehensive performance of new silicate inorganic sand cores. Furthermore, these findings reveal the mechanism by which inorganic powder accelerators affect casting sand cores during the casting process of aluminum alloy and also can be further extended to the rational design of powder components to improve the performance of inorganic sand cores for the foundry industry.

## Figures and Tables

**Table 1:** The composition of the microsilica.

SiO_2_ (wt.%)	CaO (wt.%)	Al_2_O_3_ (wt.%)	MgO (wt.%)	K_2_O (wt.%)	Others (wt.%) ^1^
98.97	0.273	0.220	0.220	0.225	0.092

^1^ Including other oxides such as Fe_2_O_3_.

**Table 2:** Bending strength of inorganic sand core cured by microsilica, the different additions of the fumed silica (0.025, 0.050, 0.075 and 0.100 wt.%) and the commercial sample.

No	Sample	Addition (wt.%)	<1 min (MPa)	1 h (MPa)	24 h (MPa)
1	commercial	/	1.810 ± 0.05197	2.991 ± 0.1319	2.794 ± 0.1013
2	microsilica	/	1.938 ± 0.08590	3.865 ± 0.09865	4.016 ± 0.02540
3	fumed silica	0.025	2.081 ± 0.1247	4.202 ± 0.3207	4.077 ± 0.1184
4	fumed silica	0.050	2.153 ± 0.1580	4.314 ± 0.2364	4.289 ± 0.1830
5	fumed silica	0.075	2.060 ± 0.1547	4.030 ± 0.2071	3.900 ± 0.2609
6	fumed silica	0.100	1.958 ± 0.1563	3.728 ± 0.3420	3.636 ± 0.2371

**Table 3:** EDS results of point scanning for inorganic sand core cured by sample with the addition of 0.050 wt.% fumed silica and microsilica.

Sample	C (wt.%)	O (wt.%)	Na (wt.%)	Al (wt.%)	Si (wt.%)	K (wt.%)
0.050 wt.% fumed silica	29.9	32.82	2.09	1.92	32.14	1.14
microsilica	/	61.19	10.93	0.18	27.49	0.21

**Table 4:** EDS results of line scanning for inorganic sand core cured by sample with the addition of 0.050 wt.% fumed silica and microsilica.

Sample	C (wt.%)	O (wt.%)	Na (wt.%)	Al (wt.%)	Si (wt.%)	K (wt.%)	Ca (wt.%)
0.050 wt.% fumed silica	4.72	59.96	9.36	0.45	25.23	0.27	/
microsilica	/	59.61	9.85	0.16	29.88	0.36	0.11

## Data Availability

The original contributions presented in the study are included in the article, further inquiries can be directed to the corresponding authors.
